# Lymphotropism of Merkel Cell Polyomavirus Infection, Nova Scotia, Canada

**DOI:** 10.3201/eid1611.100628

**Published:** 2010-11

**Authors:** Sonia Toracchio, Annette Foyle, Vojtech Sroller, Jon A. Reed, Jun Wu, Claudia A. Kozinetz, Janet S. Butel

**Affiliations:** Author affiliations: Baylor College of Medicine, Houston, Texas, USA (S. Toracchio, V. Sroller, J.A. Reed, C.A. Kozinez, J.S. Butel);; Dalhousie University, Halifax, Nova Scotia, Canada (A. Foyle);; Queen Elizabeth II Health Science Center, Halifax (A. Foyle);; Public Health Agency of Canada, Ottawa, Ontario, Canada (J. Wu)

**Keywords:** Viruses, Merkel cell polyomavirus, MCPyV, human polyomaviruses, lymphocytes, cancer, lymphotropic, chronic lymphocytic leukemia/small lymphocytic lymphoma, Canada, research

## Abstract

To test the hypothesis that Merkel cell polyomavirus (MCPyV) can infect cells of the lymphoid system, we analyzed 353 specimens, including 152 non-Hodgkin lymphomas, 44 Hodgkin lymphomas, 110 benign lymph nodes, 27 lymph nodes with metastasis, and 20 extranodal tissue samples. MCPyV DNA was detected by quantitative PCR in 13 (6.6%) of 196 lymphomas, including 5 (20.8%) of 24 chronic lymphocytic leukemia specimens, and in 11 (10%) of 110 benign lymph nodes, including 8 (13.1%) of 61 samples of reactive hyperplasia and 3 (10.3%) of 29 normal lymph nodes. Other samples were MCPyV negative. Sequence analysis of 9 virus-positive samples confirmed the identity of MCPyV; 3 viral strains were represented. Immunohistochemical testing showed that 1 T-cell lymphoma expressed MCPyV T-antigen. These findings suggest that the lymphoid system plays a role in MCPyV infection and may be a site for MCPyV persistence.

## Abstract 2

Lymphoid cells may be a site for virus persistence.

Merkel cell polyomavirus (MCPyV) was first described in 2008 (*1*) as a new human virus associated with Merkel cell carcinoma (MCC), an uncommon but aggressive form of skin cancer. Subsequent studies have reported the presence of MCPyV in 24%–100% of MCCs from patients from the United States, Germany, France, the Netherlands, and Australia (*1**–**11*). Findings of the clonal integration of MCPyV in tumor cell genomes, tumor-associated mutations in the large T-antigen (T-ag) gene, and large T-ag expression in tumors suggest that MCPyV is not only associated with MCC, but that it may be the causative agent (*1**,**12**,**13*). However, the natural reservoir of MCPyV in infected hosts remains to be identified. MCPyV DNA has been detected at low copy number in some non-MCC skin tumors, in normal tissues of skin and the gastrointestinal tract, and in a few nasopharyngeal aspirates and blood samples, including inflammatory monocytes (*1**,**5**,**6**,**11**,**14**–**16*).

Lymphocytes can disseminate viruses throughout a host and may provide sites of viral persistence. Human polyomaviruses are known to establish persistent infections in healthy persons, to undergo periodic reactivation and replication, and to cause disease in susceptible hosts. Some polyomaviruses are lymphotropic; BK virus, simian virus 40, and JC virus DNA sequences have been detected in human lymphoid tissues, blood cells, and lymphomas (*17**–**20*). Recently, Shuda et al. (*13*) reported the presence of MCPyV in a low percentage (2.2%) of hematolymphoid malignancies. In this study, we investigated the presence of MCPyV in benign lymph nodes and malignant lymphomas in patients from Canada.

## Design and Methods

### Patients and Samples

This study was approved by the Capital Health Research Ethics Board, Halifax, Nova Scotia, Canada, and by the **Baylor College of Medicine** Institutional Review Board, Houston. A total of 353 frozen specimens from various body sites were analyzed. Tissues from 196 malignant lymphomas, including 152 non-Hodgkin lymphoma (NHL) and 44 Hodgkin lymphoma (HL) samples, were retrieved from the Department of Anatomical Pathology, Queen Elizabeth II Health Sciences Center, Halifax, Nova Scotia, Canada.

All cases were diagnosed during 1994 through 2001 and classified according to World Health Organization criteria by using morphologic and immunohistochemical evaluation (*21*). The NHL samples were classified as B-cell lymphoma (n = 133), NK/T-cell lymphoma (n = 18), or were unclassified (n = 1). The HL samples were classified as classical HL (n = 41) or nodular lymphocyte predominant HL (n = 3). Also included in our study were 157 nonlymphoma frozen tissue specimens, including 110 benign lymph node biopsy specimens from healthy patients or patients with inflammatory disease, 27 lymph nodes from patients with metastatic carcinoma or melanoma, 7 biopsy specimens of inflammatory tissues, and 13 other neoplastic (non-MCC) tissue samples.

Lymphoma specimens were obtained from 96 women and 98 men; patients’ ages ranged from 15 to 88 years (mean 54.5 years). Of the 110 benign lymph nodes, 53 were from women and 57 from men, ranging in age from 17 to 85 years (mean 46 years). Lymph nodes with metastatic tumors were obtained from 16 women and 10 men, ranging in age from 22 to 86 years (mean 60.6 years). Of the 7 inflammatory tissue samples, 3 were from women and 4 were from men 19–84 years of age (mean 52 years). Finally, the non-MCC tumors were obtained from 8 men and 5 women, ranging in age from 22 to 72 years (mean 50.5 years). Data were not available for 3 patients, 2 with lymphoma and 1 with metastatic cancer. The cancers of all lymphoma patients were staged according to the Ann Arbor system (*22*).

Formalin-fixed, paraffin-embedded biopsy **specimens of skin cancers (4 MCCs** and 4 melanomas), obtained from the archives of the Department of Pathology, Baylor College of Medicine, Houston, Texas, USA, served as controls for PCR. Specimens were collected during 1998–2008.

### Immunohistochemical Analysis

The expression of MCPyV T-ag was detected by immunohistochemical analysis by using a BenchMark XT IHC (Ventana Medical Systems, Inc., Tucson, AZ, USA) system. Tissue sections on microslides were deparaffinized with xylene, hydrated in serially diluted alcohol, and endogenous peroxidase activity was quenched. The sections were then treated with slightly basic Tris-based buffer for 30 min for antigen retrieval. Sections next were incubated with CM2B4, a monoclonal antibody against MCPyV large T-ag (Santa Cruz Biotechnology, Inc., Santa Cruz, CA, USA) at a dilution of 1:250, followed by a standard polymer detection kit (diaminobenzidine) that served as the chromogen. The slides were counterstained with hematoxylin, dehydrated, and mounted for examination. A sample from a patient with MCC served as a positive control.

### DNA Extraction

DNA was extracted from frozen tissues by lysis buffer/proteinase K treatment and phenol-chloroform extraction as previously described (*20*), omitting the deparaffinization step. For the paraffin-embedded specimens, sections were deparaffinized, and DNA was extracted by using our previously described protocol (*20*). During sample processing, stringent precautions were taken to avoid cross-contamination **between** samples. The microtome was cleaned carefully, and the blade was replaced for sectioning of each tissue. Other safety measures included working within a biosafety hood located in a dedicated room free from plasmids and viruses and using a dedicated set of pipettors and barrier filter tips. In addition, a negative extraction control that lacked tissue was processed in parallel.

### Real-Time Quantitative PCR

Samples were first screened for the single-copy human RNase P gene by real-time quantitative PCR (qPCR), as described (*20**,**23*). Briefly, 5 μL of each DNA sample (undiluted and 1:10 diluted) was used in 50-μL qPCRs. This strategy detected potential PCR inhibitors in the DNA preparations, determined the human cell equivalents in each DNA sample, and normalized MCPyV viral loads to human cell numbers.

MCPyV was detected by qPCR with primers and TaqMan probe (Applied Biosystems, Foster City, CA, USA) designed to detect sequences from the unique coding region of the small tumor antigen (t-ag) gene of MCPyV. The sequences of the oligonucleotides were as follows: LT3-fwd primer 5′-AGTGTTTTTGCTATCAGTGCTTTATTCT-3′, corresponding to nt 632–659 of MCPyV350 (GenBank accession no. EU375803); LT3-rev primer 5′-CCACCAGTCAAAACTTTCCCA-3′, corresponding to nt 702–682; and fluorogenic probe 5′-FAM-TGGTTTGGATTTCCTC-MGB-3′, corresponding to nt 661–676. The pCR.MCV350 plasmid described by Feng et al*.* (*1*) was used as a positive control. Amplifications were performed with the ABI Prism 7700 Sequence Detection System (Applied Biosystems, Foster City, CA, USA) by using the following cycling parameters: 2 min at 50°C, 10 min at 95°C, followed by 40 cycles of 15 s at 95°C and of 1 min at 60°C. All PCRs were performed in duplicate. Reactions were considered positive if >10 viral genome copies/reaction were detected.

### Conventional PCR

Conventional PCR was performed on a subset of DNA samples to validate the presence of MCPyV. The primer set used was the following: MC_F2 (5′-CTCATCCTCTGGATCCAGTAGC-3′) and MC_R2 (5′-CAGAAGAGATCCTCCCAGGTG-3′) specific for a conserved region between nt positions 1142 and 1267 of the large T-ag gene of MCPyV (GenBank accession no. EU375803) and gave a 126-bp fragment. The pCR.MCV350 positive control plasmid was added to the control PCR outside the clean room after tubes containing test DNAs and negative controls (without DNA template) were closed. PCRs were performed in a GeneAmp PCR System 2700 thermal cycler (Applied Biosystems). **Thermal** cycling parameters **were** as **follows**: 94°C for 2 min, followed by 45 cycles at 94°C for 15 s, 60°C for 15 s, and 72°C for 15 s, with a final extension step at 72°C for 7 min. Amplified fragments were visualized by electrophoresis on a 3% agarose gel and stained with ethidium bromide. All PCR products were then purified by using the DNA Clean & Concentrator-5 system (Zymo Research, Orange, CA, USA) and sequenced by Lone Star Labs, Inc. (Houston, TX, USA).

### Statistical Analysis

The z score of difference for proportions was used to test for a difference between selected groups and an outcome. Fisher exact test was used to test the distribution of clinical stage of disease and 5-year survival category between MCPyV-status groups; a p value <0.05 was considered significant.

## Results

### DNA Recoveries

DNA was extracted from the specimens and screened for suitability for qPCR analysis by amplification of the cellular RNase P gene. Serial dilutions were tested to determine whether PCR inhibitors were present in the DNA samples and to select noninhibitory dilutions for MCPyV analysis. Total DNA yields ranged from 41 × 10^4^ to 403 × 10^6^ cell equivalents (median 51 × 10^6^) for the lymphoma samples, from 64 × 10^3^ to 216 × 10^6^ cell equivalents (median 29 × 10^6^) for the benign lymph node samples, from 13 × 10^5^ to 117 × 10^6^ cell equivalents (median 21 × 10^6^) for the lymph node samples with metastatic cancer, from 30 × 10^5^ to 78 × 10^6^ cell equivalents (median 39 × 10^6^) for the other inflammatory tissues, and from 16 × 10^5^ to 126 × 10^6^ cell equivalents (median 20 × 10^6^) for the other neoplastic tissues.

### MCPyV Detection in MCC

We tested 4 **MCC** and 4 melanoma **samples (fixed and paraffin-embedded)** for the presence of MCPyV by qPCR. MCPyV sequences were detected in 2 of 4 MCC samples; none were detected in the 4 melanoma samples (Table 1). MCCs contained an average of 0.29 (range 0.02–0.56) MCPyV genome copies per cell. MCPyV in 1 sample was confirmed by conventional PCR and sequence analysis. The sequence of the **amplified** fragment showed 98% similarity with the MCC350 reference sequence (GenBank accession no. EU375803) and 100% homology with MCC349 (GenBank accession no. FJ173813).

**Table 1:** MCPyV DNA from lymphoid and nonlymphoid samples, Halifax, Nova Scotia, Canada, and from MCC tissues, Houston, Texas, USA, 1994–2008*

Type of specimen	No. samples tested	No. (%) MCPyV positive
Frozen samples from Halifax, Nova Scotia, Canada		
Malignant lymphomas	196	13 (6.6)
Benign lymph nodes	110	11 (10.0)
Lymph nodes with non-MCC metastatic cancer	27	0
Other inflammatory tissues	7	0
Other neoplastic non-MCC tissues	13	0
Totals	353	24 (6.8)
Fixed tissues from Houston, Texas, USA		
MCC	4	2 (50.0)
Melanoma	4	0
*MCPyV, Merkel cell polyomavirus; MCC, Merkel cell carcinoma.		

### MCPyV Detection in Malignant Lymphomas, Lymphoid Tissues, and Other Inflammatory or Neoplastic Tissues

A total of 196 frozen malignant lymphoma samples were tested for MCPyV sequences. The classification of those samples is summarized in Table 2. Viral DNA was detected in 13 (6.6%) of the lymphomas (Tables 1, 2). As determined by qPCR, the viral copy numbers were relatively low. An average of 4.6 copies/10^4^ cells (range 0.16–27 copies/10^4^ cells, median 0.94 copies/10^4^ cells) was detected in the MCPyV-positive lymphomas. Among the lymphomas, the overall frequency of MCPyV between NHL and HL cases was similar (6.6% and 6.8%; p = 1.0) (Table 2).

**Table 2:** Presence of MCPyV in malignant lymphomas, Nova Scotia, Canada, 1994–2001*

Pathology	No. samples tested	No. (%) MCPyV positive
Non-Hodgkin lymphoma	152	10 (6.6)
B-cell lymphoma	133	8 (6.0)
Burkitt lymphoma/Burkitt cell leukemia	2	0
Chronic lymphocytic leukemia/small lymphocytic lymphoma	24	5 (20.8)
Diffuse follicular center lymphoma	1	0
Diffuse large B-cell lymphoma	52	0
Diffuse large B-cell lymphoma/T-cell/histiocyte-rich type	1	0
Extranodal marginal zone B-cell lymphoma	2	0
Follicular lymphoma	35	2 (5.7)
Lymphoblastic leukemia/lymphoma	1	0
Lymphoplasmacytic lymphoma	4	0
Mantle cell lymphoma	7	0
Posttransplant lymphoproliferative disorder, polymorphic	1	0
Splenic marginal zone lymphoma	1	0
Unclassified	2	1 (50.0)
NK/T-cell lymphoma	18	2 (11.0)
Anaplastic large cell lymphoma	8	1 (12.5)
Angiocentric T cell lymphoma (nasal type)	1	0
Angioimmunoblastic T-cell lymphoma	2	1 (50.0)
Extranodal NK/T-cell lymphoma, nasal type	2	0
Lymphoblastic lymphoma	4	0
Peripheral T-cell lymphoma	1	0
Unclassified	1	0
Hodgkin lymphoma	44	3 (6.8)
Classical Hodgkin lymphoma	41	3 (7.3)
Mixed cellularity	10	2 (20.0)
Nodular sclerosis	29	1 (3.4)
Unclassified	2	0
Nodular lymphocyte predominant Hodgkin lymphoma	3	0
All	196	13 (6.6)

*MCPyV, Merkel cell polyomavirus.

MCPyV was identified most frequently in chronic lymphocytic leukemia/small lymphocytic lymphoma (CLL/SLL) (20.8%), a tumor type known to occur in MCC patients (Table 2). Patients with CLL/SLL had a high prevalence of MCPyV compared to all other B-cell lymphomas (20.8% vs. 2.8%; p = 0.01). The virus loads in CLL/SLL were similar to those found in patients with other types of lymphoma. Characteristics of MCPyV-positive and MCPyV-negative CLL/SLL patients are shown in Table 3. None of the 24 CLL/SLL patients had MCC.

**Table 3:** MCPyV infection and characteristics of patients with chronic lymphocytic leukemia/small lymphocytic lymphoma, Nova Scotia, Canada, 1994–2001*

Characteristic	No. (%) cases	No. (%) MCPyV positive	No. (%) MCPyV negative
Patient sex			
M	16 (66.7)	2 (12.5)	14 (87.5)
F	8 (33.3)	3 (37.5)	5 (62.5)
Patient age, y			
<60	6 (25.0)	0	6 (100.0)
>60	18 (75.0)	5 (27.8)	13 (72.2)
Disease stage†			
I	9 (40.9)	1 (11.1)	8 (88.9)
II	5 (22.7)	3 (60.0)	2 (40.0)
III	0	0	0
IV	8 (36.4)	1 (12.5)	7 (87.5)
Patient survival‡			
Alive in remission	2 (15.4)	1 (50.0)	1 (50.0)
Alive with disease	6 (46.1)	0	6 (100.0)
Dead from disease	5 (38.5)	1 (20.0)	4 (80.0)
Total	24	5 (20.8)	19 (79.2)

*No patients with chronic lymphocytic leukemia/small lymphocytic lymphoma had Merkel cell carcinoma. MCPyV, Merkel cell polyomavirus. †Stage of disease for 2 MCPyV-negative patients was unknown. ‡Five-year follow-up survival information. Data were available for 13 patients, excluding 10 who had received a diagnosis within the past 5 y and one who was lost to follow-up. Disease refers to original diagnosis of either lymphoma or leukemia.

A total of 110 frozen benign lymph node samples, 27 lymph nodes with metastatic tumors, and 20 other inflammatory or neoplastic tissues were tested in parallel (Table 4). Eleven of 110 (10%) benign lymph nodes were MCPyV positive (Tables 1, 4). Viral loads in MCPyV-positive lymph nodes averaged 2.3 copies/10^4^ cells (range 0.44–6.0 copies/10^4^ cells, median 2.1 copies/10^4^ cells). Of the 11 benign lymphoid specimens positive for MCPyV DNA, 8 were among 61 (13.1%) reactive hyperplasia samples and 3 were from 29 (10.3%) normal lymph nodes. All other samples tested were negative for MCPyV (Table 4).

**Table 4:** Presence of MCPyV in benign lymph nodes, lymph nodes with metastatic cancer, and other inflammatory or neoplastic tissues, Nova Scotia, Canada, 1994–2001*

Pathologic feature	No. samples tested	No. (%) MCPyV positive
Benign lymph nodes	110	11 (10)
Acute lymphadenitis	1	0
Atypical hyperplasia	2	0
Dermatopathic lymphadenopathy	3	0
Florid follicular hyperplasia	1	0
*Mycobacterium avium–intracellulare* infection	2	0
Necrotizing granulomas	3	0
Normal lymph node	29	3 (10.3)
Reactive hyperplasia	61	8 (13.1)
Sarcoidosis	6	0
Systemic mast cell disease	1	0
Toxoplasmosis	1	0
Lymph nodes with metastatic tumors	27	0
Carcinoma	25	0
Melanoma	2	0
Other inflammatory tissues	7	0
Chronic sialadenitis	1	0
Follicular bronchiolitis	1	0
Hemophagocytic syndrome, spleen	1	0
*Helicobacter pylori–*associated gastritis	1	0
Hemochromatosis, liver	1	0
Hyperplasia and chronic perifolliculitis, skin	1	0
Interstitial pneumonitis	1	0
Other neoplastic tissues	13	0
Mixed mullerian tumor	1	0
Neurofibroma	1	0
Sarcoma	4	0
Schwannoma	2	0
Thymoma	5	0

*MCPyV, Merkel cell polyomavirus.

Sequence analysis confirmed the qPCR results. Conventional PCR with MCPyV-specific primers was performed on 9 viral DNA-positive samples (4 lymphoma, 5 reactive hyperplasia). DNA amplification products of the expected size (126 bp) from the large T-ag gene were obtained, and **sequence analysis confirmed the identity of MCPyV. Three of 4 MCPyV-positive lymphoma samples shared 100% sequence homology with strain MCC344 (GenBank accession no. FJ173807), whereas sequences of the remaining sample were identical to those of MCC349 (GenBank accession no. FJ173813). Among the 5 MCPyV-positive reactive hyperplasia samples, 4 had 100% homology to strain MCC344 and 1 to strain MCC339 (GenBank accession no. EU375804).**

### Expression of MCPyV T-antigen

A series of 17 lymphoid specimens (**7 of which were positive for MCPyV DNA by PCR),** consisting of 11 lymphomas and 6 reactive hyperplasia samples, **were tested for expression of MCPyV T-ag. Immunohistochemical staining with the CM2B4 monoclonal antibody was carried out without knowledge of the PCR results. One sample, classified as an angioimmunoblastic T-cell lymphoma, expressed detectable T-ag in scattered lymphocytes (**Figure**). This sample was positive for MCPyV DNA by PCR. The remaining samples tested were negative for T-ag expression by immunohistochemical test.**

**Figure:**
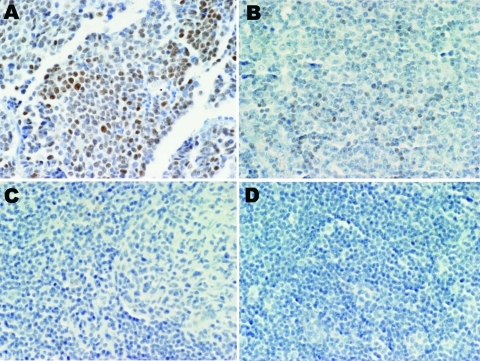
Merkel cell polyomavirus (MCPyV) large T-antigen (T-ag) expression in human tissues. A) Merkel cell carcinoma stained with CM2B4 antibody as a positive control; MCPyV T-ag was detected. B) Expression of MCPyV T-ag in small lymphocytes in an MCPyV DNA–positive angioimmunoblastic T-cell lymphoma, stained with CM2B4. C) MCPyV DNA–positive reactive lymphoid hyperplasia sample reacted with CM2B4; no T-ag was detected. D) MCPyV DNA-negative chronic lymphocytic leukemia/small lymphocytic lymphoma sample stained with CM2B4; no T-ag was detected. Original magnification ×40.

### Clinical Follow-Up

**Five-year clinical follow-up** information was available for 114 of 196 patients who had malignant lymphoma. Of those patients not included in follow-up analysis, 14 had died from other causes, 47 had received a diagnosis of lymphoma within the past 5 years, and 21 were lost to follow-up. Among the 114 patients with a 5-year follow-up, 49 had died of the disease and 65 were alive (24 with lymphoma and 41 without). Analyses showed no difference in the distributions among survival categories relative to MCPyV status (Table 5). Of the MCPyV-positive patients for whom follow-up data were available, 3 (42.9%) of 7 were alive and in remission compared with 38 (35.5%) of 107 MCPyV-negative patients. There were 3 (42.9%) deaths among the MCPyV-positive group and 46 (43.0%) deaths among the MCPyV-negative group (p = 0.88).

**Table 5:** Clinical outcomes for lymphoma patients over a 5-year period, Nova Scotia, Canada*

Characteristics	No. (%) MCPyV positive	No. (%) MCPyV negative	p value
Disease stage†			
I	2 (28.6)	35 (35.4)	
II	2 (28.6)	14 (14.1)	
III	0	11 (11.1)	
IV	3 (42.9)	39 (39.4)	0.62
Patient survival			
Alive in remission	3 (42.9)	38 (35.5)	
Alive with disease	1 (14.3)	23 (21.5)	
Died from disease	3 (42.9)	46 (43.0)	0.88

*The 5-year period refers to the time frame for each patient from time of diagnosis until follow-up 5 years later. MCPyV, Merkel cell polyomavirus. †Clinical stage of disease at time of lymphoma diagnosis; information was not available for 8 MCPyV-negative patients.

## Discussion

This study describes the presence of MCPyV DNA in benign lymph nodes and malignant lymphomas in specimens from patients living in Canada. MCPyV was detected in 6.6% of lymphomas and in 10% of nonneoplastic lymph node samples. These results, together with those of Shuda et al. (*13*) and Mertz et al. (*14*), support the hypothesis that lymphocytes and monocytes may serve as a tissue reservoir for MCPyV infection. Because serologic assays have indicated that MCPyV primary infections frequently occur in children (*24**–**26*), we favor the interpretation that the MCPyV genomes detected in the adult lymphoid tissues reflect the presence of persistently infected cells. Only 1 specimen among MCPyV DNA–positive samples tested expressed T-ag, which suggests that most infected lymphoid cells are not producing detectable levels of viral protein. However, because MCPyV DNA copy numbers in the samples were low, a few antigen-expressing cells in the tissues may have escaped detection in the immunohistochemical assays.

The data from this study do not suggest that MCPyV caused the lymphoid tumors that were virus positive. However, more comprehensive studies are necessary to exclude the possibility that MCPyV may have lymphomagenic potential under certain conditions. An observation of interest was the presence of MCPyV in 5 (20.8%) of 24 CLL/SLL cases. CLL is a type of leukemia that is now regarded as being identical to SLL (*27*). The most recent World Health Organization classification scheme for hematopoietic malignancies considers CLL and SLL to be different manifestations of the same disease and combines these entities into 1 disease category (CLL/SLL) (*21*). Some studies have found that CLL co-exists with MCC, making the association rare but well recognized (*28**–**34*). CLL and MCC are age-related with an increased risk in those >60 years of age. Koljonen et al. (*35*) recently showed that MCPyV DNA is frequently present in MCCs that occur in CLL patients. The basis for the association between CLL and MCPyV is unclear. The link may be coincidental or may reflect some influence of the MCPyV-infection process.

The present study provides evidence of the presence of MCPyV in samples of reactive lymphoid hyperplasia. (Reactive lymphoid hyperplasia refers to a benign, reversible enlargement of the lymph node as a consequence of proliferation of some or all of its cellular components.) This is a normal response of the lymph nodes to an antigenic stimulus, such as infection or inflammation. Viruses, e.g., **Epstein-Barr virus,** induce reactivity of lymphoid cells in lymphoid tissues from healthy persons (*36*). In our study, 8 (13.1%) of 61 reactive hyperplasia specimens were shown to harbor MCPyV DNA at low copy number. Whether MCPyV infection prompted those cases of reactive hyperplasia is unknown.

In conclusion, our findings of the presence of MCPyV in malignant lymphomas, reactive hyperplasia, and normal lymph nodes support the hypothesis that MCPyV is lymphotropic. Our findings also suggest that the lymphoid system plays a role in MCPyV infection and may be a site for MCPyV persistence.
